# Transcriptome sequencing reveals the differentially expressed lncRNAs and mRNAs in response to cold acclimation and cold stress in *Pomacea canaliculata*

**DOI:** 10.1186/s12864-022-08622-5

**Published:** 2022-05-19

**Authors:** Qi Xiao, Youfu Lin, Hong Li, Yu Chen, Wei Wei, Peng Li, Lian Chen

**Affiliations:** 1 Co-Innovation Center for Sustainable Forestry in Southern China, College of Biology and the Environment, Nanjing Forestry University, Nanjing, 210037 China; 2 Jiangsu Key Laboratory for Biodiversity and Biotechnology, College of Life Sciences, Nanjing Normal University, Nanjing, 210023 China; 3 College of Life Sciences, Chemistry and Chemical Engineering, Jiangsu Second Normal University, Nanjing, 210013 China

**Keywords:** *Pomacea canaliculata*, Cold acclimation, Cold stress, Transcriptome, lnc RNA

## Abstract

**Background:**

Tolerance of low temperature has a significant impact on survival and expansion of invasive snail *Pomacea canalicuata*. Cold acclimation can enhance cold tolerance of *Pomacea canalicuata*. To elucidate the molecular mechanism of *P. canaliculata*’s responses to cold acclimation and cold stress, a high-throughput transcriptome analysis of *P. canaliculata* was performed, and gene expression following artificial cold acclimation and then cold stress at 0 °C for 24 h was compared using RNA sequencing.

**Results:**

Using the Illumina platform, we obtained 151.59 G subreads. A total of 5,416 novel lncRNAs were identified, and 3166 differentially expressed mRNAs and 211 differentially expressed lncRNAs were screened with stringent thresholds. The potential antisense, cis and trans targets of lncRNAs were predicted. Kyoto Encyclopedia of Genes and Genomes enrichment analysis showed that many target genes were involved in proteasome, linoleic acid metabolism and retinol metabolism under cold acclimation. The lncRNA of *P. canaliculata* could participate in cold acclimation by regulating the expression of *E3 ubiquitin protein ligase*, *26S proteasome non-ATPase dependent regulation subunit*, *glutathione S-transferase, sodium/glucose cotransporter* and *cytochrome P450*.

**Conclusions:**

These results broaden our understanding of cold acclimation and cold stress associated lncRNAs and mRNAs, and provide new insights into lncRNA mediated regulation of *P. canaliculata* cold acclimation and cold stress response.

**Supplementary Information:**

The online version contains supplementary material available at 10.1186/s12864-022-08622-5.

## Background

*Pomacea canaliculata* is a pantropical species of invasive freshwater gastropods notorious for damaging wetlands and agriculture. Whether an introduced species becomes invasive is dependent on both the characteristics of the niche and the biological traits of the species [1, 2]. Niche-based characteristics include climate suitability [3], resource availability [4] and presence of potential competitors [5]. Trait-based characteristics of the species include life span, growth rate, fecundity, dispersal ability, dietary spectrum, genetic adaptation, and environmental tolerance [6–8]. For *P. canaliculata*, the strong abilities to feed [9], grow and reproduce [10] are known to be associated with their strong invasion. In addition to the characteristics described above, tolerance of low temperature is another key characteristic for *P.canaliculata* to expand in temperate East Asia and tropical Southeast Asia [11, 12].

For many invasive species, the climate of the new habitat is the major factor determining colonization success. Species that cannot tolerate or adapt to local climate will disappear shortly after initial invasion [13]. The natural range of *P. canaliculata* consists of the Lower Paraná, Uruguay, and La Plata basins [14]. For *P. canaliculata,* growth, feeding and crawling are completely suppressed at temperature below 10 °C [15]. In temperate Japan, *P. canaliculata* inhabiting paddy fields exhibit high mortality in winter, with levels ranging from 65 to 99.8%. The expansion range of *P. canaliculata* in East Asia has been restricted by cold winter weather [11, 16, 17].

There are two ways for *P. canaliculata* to resist cold climates. Behaviorally, snails can bury themselves into soil or under rice straw when the paddy fields are drained before harvest. The temperatures under the straw generally remain above 0 °C, even when the soil surface temperatures drop below -5 °C [18]. Another approach is the physiological enhancement of cold tolerance. *P. canaliculata* in paddy fields in temperate Japan increase their cold tolerance before the onset of winter [18]. Gradually decreasing temperatures (cold-acclimation) serve as the main environmental cue for *P. canaliculata* to enhance cold tolerance. After cold acclimation, *P. canaliculata* can enhance cold hardiness by accumulating low-molecular-weight compounds in their body, such as glucose, glycerol, glutamine and carnosine [13, 19]. In general, low-molecular-weight compounds can act as cryoprotectants, and their presence can decrease the supercooling points, prevent protein denaturation and reduce cuticular water loss by binding water [20]. Although previous studies showed that the expression of glycerol kinase (GK), heat shock protein 70 (HSP70), Na^+^/K^+^-ATPase (NKA), and glycerol-3-phosphate dehydrogenase (GPDH) genes is related to the cold hardiness of *P. canaliculata* [21], few studies focused on the molecular mechanism of increased cold hardiness.

With rapid development in high-throughput transcriptome sequencing technologies, the identification of differentially expressed genes (DEGs) triggered by cold response has been performed in a large number of species, helping to discover numerous non-coding RNA genes [22]. These efforts have led to a better understanding of the molecular mechanisms involved in the adaptation of *P. canaliculata* to low temperatures. LncRNAs (Long non-coding RNAs) are over 200 nucleotides in length, have a low ability to code proteins, and account for the vast majority of ncRNAs [23]. LncRNAs have been identified to regulate gene expression in the close (*cis*-acting) or distant (*trans*-acting) regions of the genome through diverse mechanisms, such as promoter activity modification via nucleosome repositioning, DNA methylation, histone modification, accessory protein activation/gathering/transportation, repression, and epigenetic silencing [24, 25]. Numerous recent studies have indicated that lncRNAs can enhance the biological stress resistance by regulating expression of functional genes [26–28]. The lncRNA *SVALKA* was identified as a negative regulator of expression of C-repeat/dehydration-responsive element binding factors (CBFs) and plant freezing tolerance [29–31]. Over-expression of *TUG1* lncRNA (*TUG1*, taurine up-regulated gene 1) protects against cold-induced liver injury in *Mus musculus* by inhibiting apoptosis and inflammation [32]. It has also been reported that lncRNAs are involved in response to cold stress in Wistar rats, giant panda (*Ailuropoda melanoleuca*) sperm, grapevine (*Vitis vinifera* L.), and pear (*Pyrus pyrifolia* 'Huanghua') [33–36].

The participation of lncRNAs in cold acclimation and under cold stress of *P. canaliculata*, however, remains unclear. In this study, we explored the response of lncRNAs and mRNAs to cold acclimation and cold stress in *P. canaliculata* using next-generation sequencing (NGS) by performing RNA-seq. This study provides insight in understanding the molecular mechanism underlying cold response of *P. canaliculata*, and shed light on the potential roles of lncRNAs in cold acclimation and cold stress-responsive *P. canaliculata*.

## Results

### Survival rate of *P.canaliculata*

When the experiment ended, we put the remaining individuals of the cold acclimation group and the control group into two boxes with water at room temperature respectively. We observed that all individuals were active within 30 min in the cold acclimation group, but there were three snails died in the control group. The survival rates of the cold acclimation group and the non-cold acclimation group were 100.00 and 96.67%, respectively in the present study. These results were similar to previous study on *P. canaliculata*. Almost all *P. canaliculata* survived at 0 °C for 2 or 5 days whether or not they had undergone cold acclimated, the survival rates of the cold acclimation group and the non-cold acclimation group were 98.3 (± 2.9) % and 96.7 (± 5.8) % respectively [13].

### Sequencing and assembly

As the main immune organ of gastropod mollusks, the hepatopancreas plays an important role in the organism’s resistance to biotic and abiotic threats [37]. Hepatopancreas samples from 12 snails were subjected to transcriptome sequencing and data analysis. The sequencing data of raw reads ranged from 11.90 to 13.31 Gb. The average Phred score Q30 of clean reads varied from 91.09 to 93.93%. The filtered reads were subsequently compared with the *P. canaliculata* reference genome [38], and the mapped reads from the 12 libraries covered 81.33–87.00% of the *P. canaliculata* genome (Supplement Table S1). All the sequences have been uploaded to the NCBI SRA database under the BioProject identifier PRJNA788380.

**Fig. 1:**
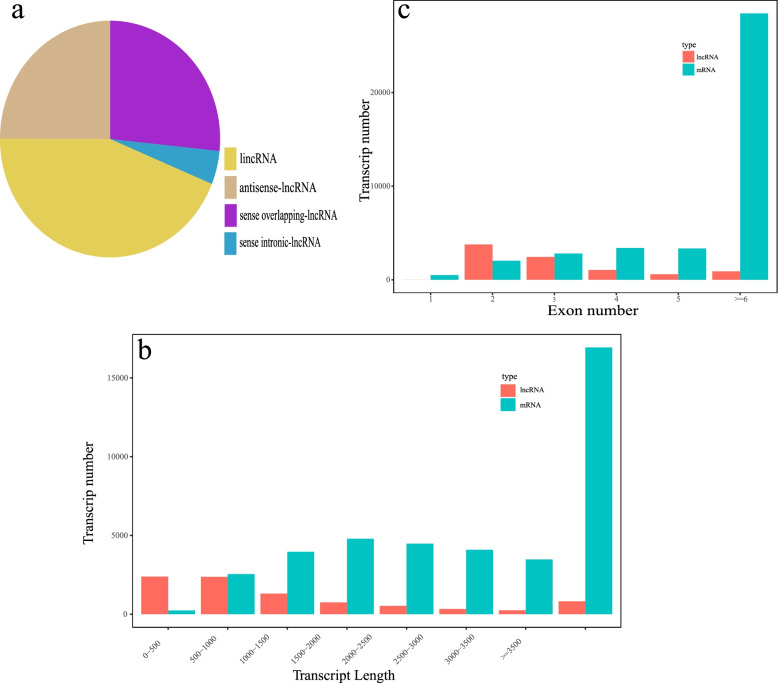
Characteristics of cold-responsive lncRNAs in *P. canaliculata* hepatopancreas. **a** four types of lncRNAs (lincRNA, antisense-lncRNA, sense overlapping-lncRNA and sense intronic-lncRNA) in hepatopancreas. **b** Numbers of mRNA and lncRNAs with different length. **c** Numbers of mRNA and lncRNAs containing different exon numbers

A total of 18,362 transcripts were assembled, and 5,416 novel lncRNAs were identified, 1,545 of which were classified as intergenic lncRNAs (also known as lincRNAs, 28.50%), 1,581 of which were classified as antisense lncRNAs (29.20%) and 2,289 of which were classified as sense overlapping lncRNAs (42.30%). The basic genomic features of these lncRNAs were characterized. Compared with protein coding genes, lncRNAs were typically 200–1500 bp in length and composed of fewer exons, with most containing two or three exons (Fig. 1). These results suggest that the majority of lncRNAs in *P. canaliculata* have shorter lengths and fewer exons compared to protein coding genes (Fig. 1).

### Differentially expressed genes and lncRNAs under cold stress

**Fig. 2:**
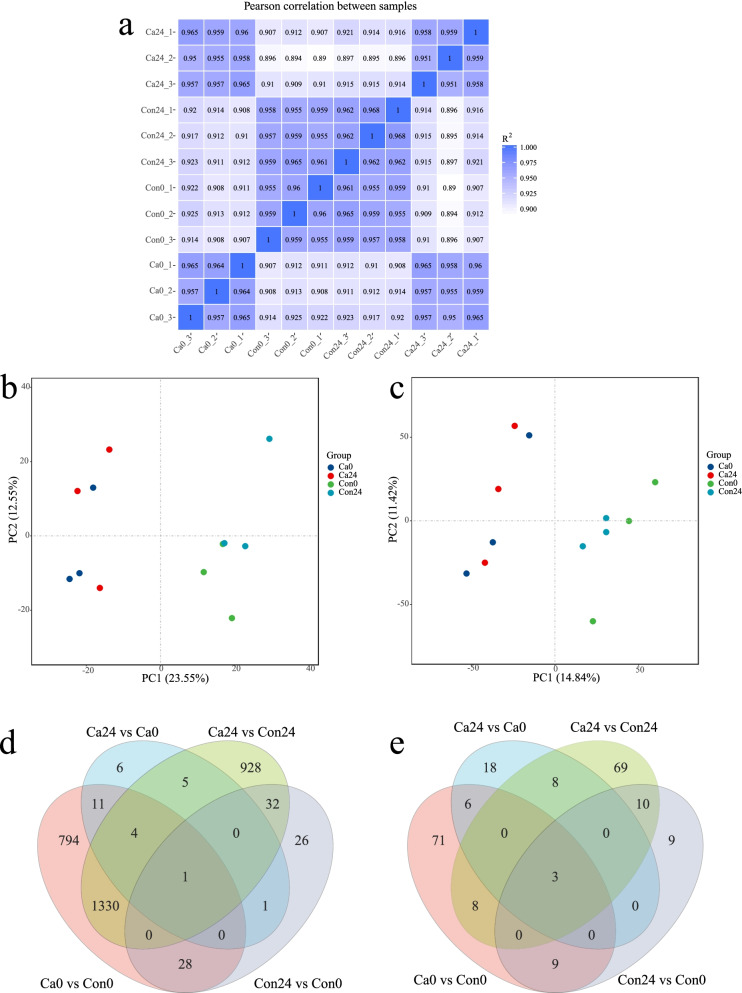
Heat map of correlation coefficient 12 samples, principal component analysis (PCA) and venn plot for lncRNA and coding-gene expressions. **a** Heat map of correlation coefficient; **b** PCA for the coding-gene expressions for *P. cancaliculata* in two time points between the cold acclimation group and the control group; **c** PCA for the lncRNAs for *P. cancaliculata* in two time points between the cold acclimation group and the control group; **d** Venn plot for the protein coding genes of *P. cancaliculata* in two time points between the cold acclimation group and the control group; **e** Venn plot for lncRNAs for *Pomacea cancaliculata* in two time points between the cold acclimation group and the control group

Expression levels were analyzed using the edgeR software to screen differentially expressed genes and lncRNAs (DELs) between the cold acclimation group and the control group. The three sample-replicates showed a high degree of concordance in gene expression (*R*^2^ correlation > 0.95, Fig. 2a). Unsupervised principal component analysis (PCA) on the transcriptomes revealed a clear discrimination between the cold acclimation group and the control group, indicating location-specific transcriptional identities (Fig. 2b, c). For Ca0 vs Con0, there were 2,168 DEGs identified, 1,129 were up-regulated and 1,039 were down-regulated. For Ca24 vs Con24, 2,300 DEGs were identified, 1,328 up-regulated and 972 down-regulated. Totally 28 (Ca24 vs Ca0) and 88 (Con24 vs Con0) significant DEGs were identified, including 18 up-regulated and 10 down-regulated genes altered between Ca24 and Ca0 and 33 up-regulated and 55 down-regulated genes altered between Con24 and Con0 (Table 1, Fig. 2d).

**Table 1:** Number of differential expression mRNA and lncRNA in experimental and control group of *Pomacea canaliculata*

Comparison groups	Ca0 vs Con0	Ca24 vs Con24	Con24 vs Con0	Ca24 vs Ca0
Up-regulated mRNA	1129	1328	33	18
Down-regulated mRNAs	1039	972	55	10
Up-regulated lncRNAs	57	60	14	18
Down-regulated lncRNAs	40	38	17	17
Co-expression target genes	637	875	169	135
Co-location target genes	1195	1085	455	439

Con0: 0 °C cold treatment of control group at 0 h; Con24: 0 °C cold treatment of control group at 24 h; Ca0: 0 °C cold treatment of experimental group at 0 h; Ca24: 0 °C cold treatment of control group at 24 h; Co-expression: the lncRNA function was predicted by mRNA genes associated with its expression; Co-location: the lncRNA function was predicted by mRNA genes associated with its location

We identified 195 DELs between the cold acclimation group and the control group, including 57 up-regulated and 40 down-regulated lncRNAs after cold acclimation (Ca0 vs Con0) and 60 up-regulated and 38 down-regulated lncRNAs after cold stress for 24 h (Ca24 vs Con24). A total of 35 DELs were identified after cold stress in cold acclimation group (Ca24 vs Ca0), including 18 up-regulated and 17 down-regulated lncRNAs. In control group (Con24 and Con0), 31 DELs were identified after cold stress (Table 1, Fig. 2e).

### Gene Ontology (GO) and Kyoto Encyclopedia of Genes and Genomes (KEGG) enrichment analysis of DEGs

**Fig. 3:**
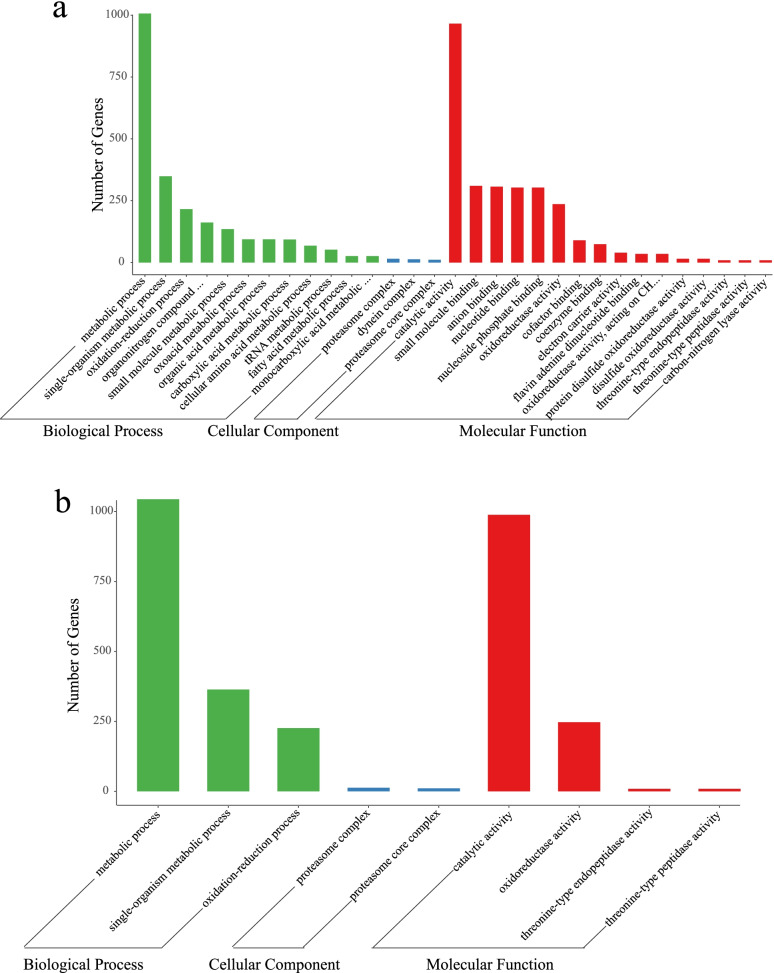
The GO enrichment of DEGs which associated with cold acclimation in *P. canaliculata.* **a** cold acclimation group and control group after cold acclimation ended (Ca0 vs Con0). **b** cold acclimation group and control group after 24 h of cold stress at 0 °C (Ca24 vs Con24)

**Fig. 4:**
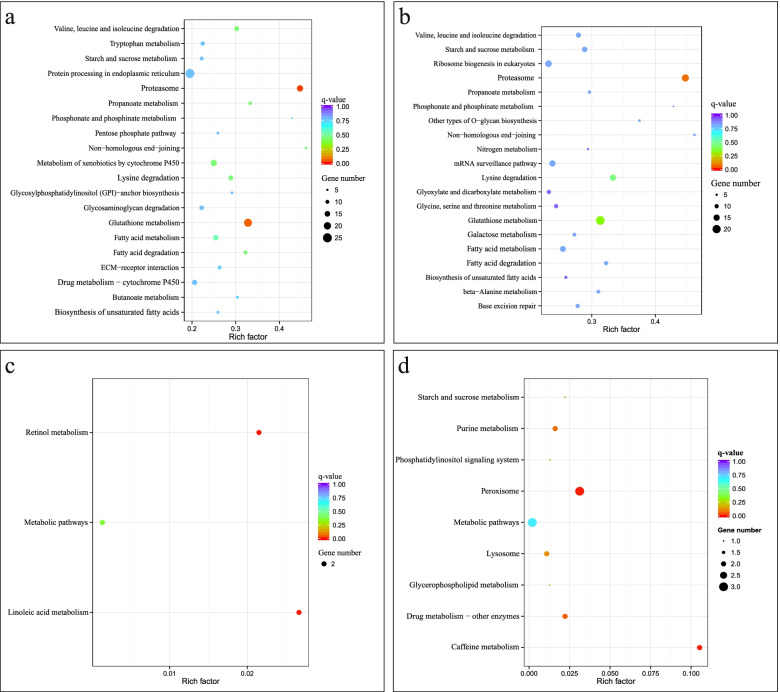
KEGG enrichment analysis of DEGs between in *P. canaliculata* **a** Ca0 vs Con0. **b** Ca24 vs Con24. **c** Ca24 vs Ca0. **d** Con24 vs Con0

To identify the genes that exhibiting significant differences between the cold acclimation group and the control group, a pairwise comparison was conducted and significance was determined using edgeR software. The DEGs that are potentially associated with cold acclimation in *P. canaliculata* were analyzed based on GO enrichment, and the results are shown in Fig. 3. After cold acclimation (Ca0 vs Con0), the DEGs were significantly enriched in 31 GO terms, among which, 12 GO terms corresponded to biological processes, 3 to cellular components, and 16 to molecular functions (Fig. 3a). For the biological processes, “metabolic process” was the most strongly represented GO term, followed by “single-organism metabolic process”. A number of genes also belonged to interesting categories, such as “cellular amino acid metabolic process”, “fatty acid metabolic process” and “organonitrogen compound metabolic process”, which may participate in cold resistance and adaptation of *P. canaliculata*. The “catalytic activity” was highly represented among the molecular functions that the genes were involved in. A total of 2,168 genes were classified into 121 KEGG functional categories, which were significantly enriched in proteasome and glutathione metabolism KEGG pathways (Fig. 4a, Table S4).

DEGs were associated with 9 GO terms under cold stress between cold acclimation group and control group (Ca24 vs Con24). The top two most significant annotated GO biological process pathways were “metabolic process” and “catalytic activity” (Fig. 3b). KEGG enrichment analysis showed that a total of 2300 DEGs were enriched in 118 KEGG pathways involved in the proteasome, glutathione metabolism, lysine degradation, and non-homologous end-joining. Starch and sucrose metabolism also contribute to the degradation of fatty acids. These DEGs were significantly enriched in proteasomal metabolic pathways (Fig. 4b). A large number of enriched terms were involved in metabolic process, catalytic activity and ion binding.

There was no significant GO term enrichment for DEGs in cold acclimation group (Ca24 vs Ca0). However, these DEGs were significantly enriched in linoleic acid metabolism and retinol metabolism pathways (Fig. 4c).

The DEGs identified in control group (Con24 vs Con0) exhibited no significant GO term enrichment. The 88 DEGs were involved in 9 KEGG pathways. They were significantly enriched in caffeine metabolism, peroxisome, drug metabolism and purine metabolism KEGG pathways (Fig. 4d).

### Validation of RNA-Seq results with qRT-PCR

**Fig. 5:**
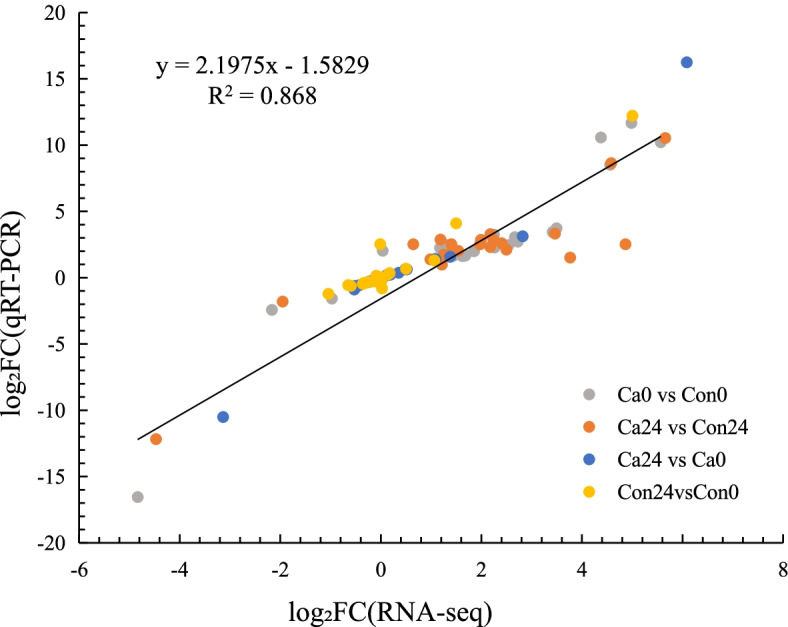
Correlation analysis of RNA-Seq and RT-qPCR in *P. canaliculata*. 22 candidate DEGs and DELs were measured by qPCR. The regression indicated a close correlation between methods (*r*^2^ = 0.868)

A total of 13 DEGs related to cold adaptation in different signaling pathways and 9 DELs were selected to validate the gene expression using qRT-PCR. The mRNA/lncRNA expression changes were similar to those identified using RNA-seq. These findings indicate that the RNA-seq results are reliable and could be used for bioinformatic analysis (Fig. 5 and Fig. S1).

### Functional prediction of long non-coding RNAs

**Fig. 6:**
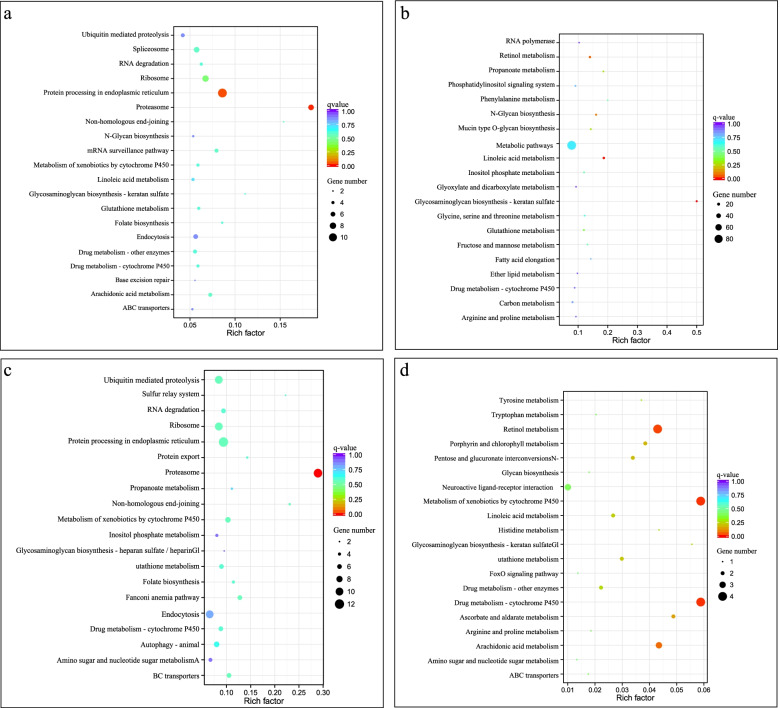
KEGG enrichment analysis of co-expressed target genes of DELs in *P. canaliculata*. *“*Rich factor” means that the ratio of the number of the genes in the specific subcluster and the number of genes annotated in this pathway. The greater the rich factor, thegreater the degree of enrichment. **a** Ca0 vs Con0; **b** Con24 vs Con0; **c** Ca24 vs Con24; **d** Ca24 vs Ca0

To understand the functional roles of the identified lncRNAs, we next explore the targets of lncRNAs. The co-expressed target genes of DELs after cold acclimation (Ca0 vs Con0) were not significantly enriched in any specific GO entries, however, they were significantly enriched in KEGG pathways associated with the proteasome and protein processing in the endoplasmic reticulum KEGG pathways (Fig. 6a). In control group (Con24 vs Con0), the co-expressed target genes of DELs were not significantly enriched in any GO entries or KEGG pathways (Fig. 6b). Co-expressed target genes of DELs exhibited no significant enrichment for any specific GO entries after cold stress (Ca24 vs Con24). KEGG enrichment analysis showed that the co-expressed target genes were enriched for the proteasome pathway (Fig. 6c). The co-expressed target genes of DELs in cold acclimation group (Ca24 vs Ca0) were significantly enriched in serine-type endopeptidase activity, serine-type peptidase activity and serine hydrolase activity and peptidase activity, acting on L-amino acid peptides under GO entries of molecular function (Table S5). They were also significantly enriched in drug metabolism-cytochrome P450, metabolism of xenobiotics by cytochrome P450 and retinol metabolism KEGG pathways (Fig. 6d).

**Fig. 7:**
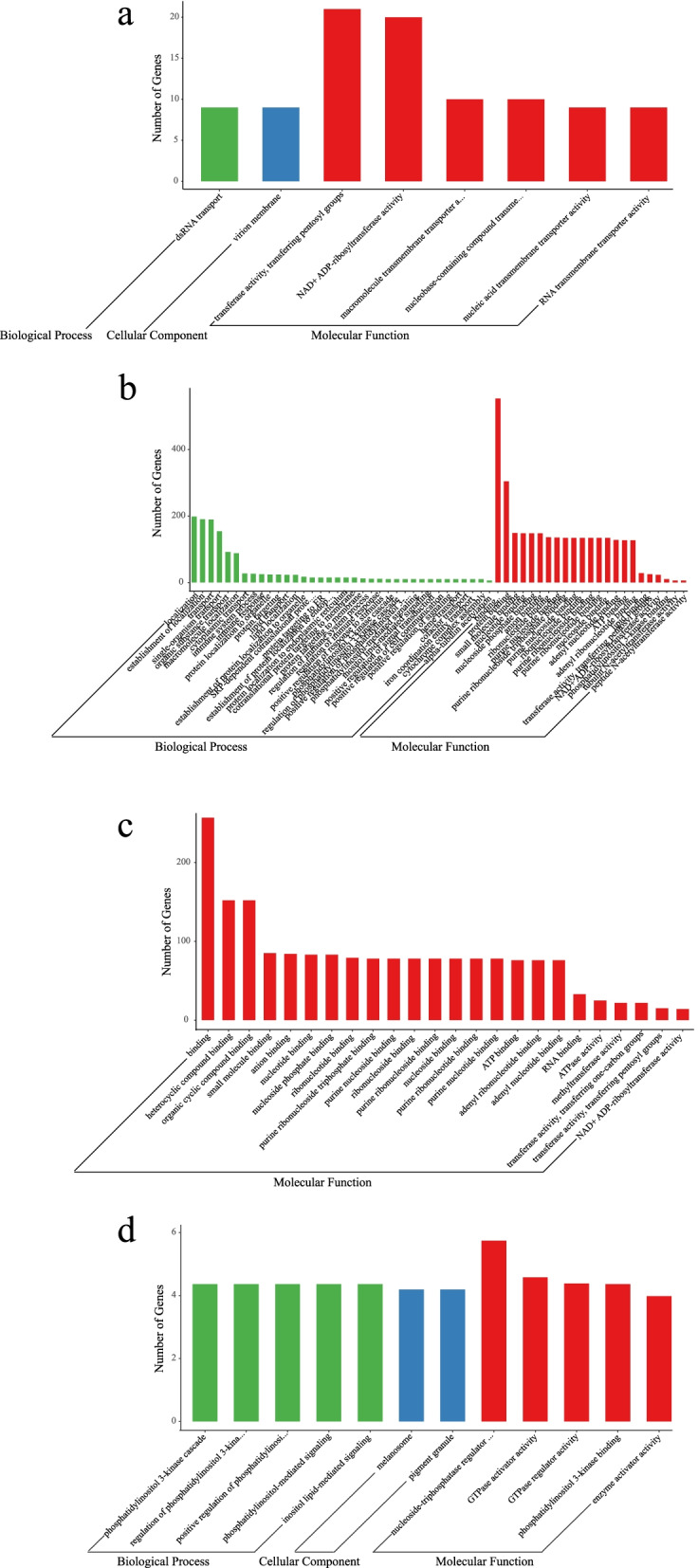
Gene Ontology (GO) enrichment analysis of co-location target genes of DELs of *P. canaliculata*. **a**: Ca0 vs Con0. **b** Ca24 vs Con24. **c** Ca24 vs Ca0. **d** Con24 vs Con0

After cold acclimation (Ca0 vs Con0), co-localized target genes of DELs were significantly enriched in 8 GO terms (Fig. 7a), with no significant enrichment found for any specific KEGG pathways. GO enrichment analysis of co-localized target genes of DELs from Ca24 vs Con24 was similar to that of Ca0 vs Con0, but more DEL-associated genes were significantly enriched in binding, protein binding as well as other binding under the molecular function category (Fig. 7b). Co-localized target genes of DELs in Ca24 vs Ca0 were significantly enriched in the 24 molecular function GO terms (Fig. 7c). Most DEL-associated genes of Ca24 vs Ca0 were significantly enriched in ATPase activity, methyltransferase activity, transferase activity, transferring one-carbon groups, binding, heterocyclic compound binding, organic cyclic compound binding, small molecule binding as well as other binding under the molecular function category (Fig. 7c). KEGG enrichment analysis showed that these genes were significantly enriched in linoleic acid metabolism and retinol metabolism pathways (Fig. S2). In control group (Con24 vs Con0), co-localized target genes of DELs were significantly enriched in 12 GO terms, such as nucleoside-triphosphatase regulator activity, GTPase activator activity and GTPase regulator activity (Fig. 7d).

## Discussion

Extreme temperatures often negatively affect both the survival of ectothermic animals and their biological functions, including reproduction, respiration, digestion, and excretion [39]. In order to reduce the negative effects of temperature on their performances, ectotherms are capable of modulating thermal tolerance over their lifetimes through a range of physiological adjustments triggered by pre-exposure to sub-lethal temperatures [40]. Low temperature acclimation involves a large suite of molecular and physiological variations [41]. In this study, we identified candidate mRNAs and lncRNAs in the *P. canaliculata* by simultaneously evaluating their differential expression patterns and correlations between the expression levels of coding and noncoding genes in response to cold stress. These lncRNAs share many characteristics of their eukaryotic counterparts: such as shorter length, fewer exons, and lower expression compared with mRNAs [42]. In our study, the lncRNA transcripts were relatively short with few exons and low levels of expression in comparison to protein coding mRNA transcripts (Fig. 1). These characteristics were also observed in *Crassostrea gigas* [43], *Haliotis discus hannai* [44] and other molluscs. We identified four aspects of *P. canaliculata* altered by cold acclimation and low temperature, which will be our focus of future work. According to the RNA-seq results, the DEGs and DELs in response to cold acclimation and cold stress related to glucose metabolism, ubiquitin–proteasome system, antioxidant system, and lipid metabolism were selected and the most important parts of them were discussed.

### Candidate cold-resistance genes related to glucose metabolism

*P. canaliculata* enhances cold hardiness by increasing glycerol and glucose content in the body [19]. In the present study, expression of *endoglucanase A, endoglucanase E-4* and *maltase-glucoamylase* genes increased in the cold acclimation group. Expression of these genes may provide additional glucose molecules for use as raw materials or increase energy of other metabolites by facilitating polysaccharide breakdown in the digestive tract. Expression of the *phosphorylase B kinase gamma-catalytic chain* gene may promote glycogen breakdown and accumulation of glucose and glycerol in *P. canaliculata*. Glycogen breakdown in *Dryophytes chrysoscelis* is transcriptionally promoted by increased expression of *phosphorylase B kinase gamma-catalytic chain* under freezing conditions [45]. The *hexokinase type 2* gene is involved in the glycolytic pathway and may play an important role in increasing glucose content [46]. Its expression was up-regulated in the cold acclimation group after 24 h at 0 °C, consistent with previous studies in goldfish (*Carassius auratus* L) [47]. Additionally, the decreased expression of *UTP-glucose-1-phosphate uridylyltransferase* and *glycogenin-1* involved in glycogen synthesis may inhibit metabolic pathways related to synthesis of glucose and glycogen. The glucose transport in purified brush boarder membrane vesicles from hepatopancreatic epithelial cells of *Panaeus japonicas* and *Homarus americanus* is carrier-mediated via a *sodium/glucose cotransporter* transport protein [48]. The increased expression of *sodium/glucose cotransporter* may play an important role in glucose transport in *P. canaliculata* during cold acclimation. *LncR112554377* can regulate the expression of *sodium/glucose cotransporter* through *cis* action, and this lncRNA may be involved in glucose transport.

### Ubiquitin–proteasome system

Ubiquitin–proteasome system (UPS) is the main protein degradation pathway, responsible for 80–90% of protein degradation in cells [49]. The UPS pathway involves two steps: first, misfolded or damaged proteins are labeled with multiple ubiquitin molecules, and then the labeled proteins are degraded by the 26S proteasome complex [50]. Compared to the control group, the E3 ubiquitin protein ligase *HECTD1*, *RNF19B* and *RNF34* were up-regulated in the cold acclimation group. The DEGs were also found to be significantly enriched in the proteasome pathway, and the expression of several genes involved as structural components of the 26S proteasome, including *26S proteasome non-ATP-dependent regulatory subunits 3*, *7*, *12* and *13,* significantly increased. The expression of *lncRNNA112553564* was significantly up-regulated in Ca0 and Ca24 conditions, with log_2_ fold changes of 7.12 and 5.48, respectively. This lncRNA can regulate the expression of *26S proteasome non-ATPase dependent regulation subunit 3*, *12* and *14* genes through *trans*-action. These results suggest that some lncRNAs regulate 26S proteasome genes to reduce the damage caused by cold stress.

### Antioxidant system-related genes

A substantial amount of reactive oxygen free radicals are produced during temperature stress [51]. In order to alleviate the pressure caused by temperature stress, factors associated with antioxidant activity, such as superoxide dismutase (SOD) and glutathione peroxidase (GSH-Px), increased [52]. Many aquatic animals enhance their antioxidant enzyme capacity by increasing their glutathione content [53]. We found that, after cold acclimation, DEGs were enriched in glutathione metabolism pathway, and the reduced form of glutathione could maintain the reductive state of the intracellular chambers and provide electrons for glutathione S-transferase (GST) and other enzymes. GST plays an important role in biological and abiotic stress responses by reducing oxidative damage caused by reactive oxygen species [54]. After cold acclimation, glutathione synthetase, the glutamate-cysteine ligase catalytic subunit involved in the first step of glutathione synthesis, *GST* subfamily omega and mu genes were up-regulated. Cytosol-aminopeptidase, which is involved in glutathione degradation, *glutathione hydrolase 1 proenzyme* and *glutathione hydrolase-like Ywrd proenzyme* were down-regulated. At the same time, when compared with Con0, the expression patterns of these genes were almost unchanged in Con24. These results suggest that cold acclimation induces synthesis of glutathione and GST, which can reduce the oxidative damage caused by cold stress. Meanwhile, cold-acclimation-induced lncRNA up-regulation is involved in GST synthesis. Prediction of lncRNA target genes indicated that *lncRNA112567329* could regulate three GST omega subfamily members through cis action. *LncRNA112568988*, *lncRNA112559114* and *lncRNA112570305* could regulate the expression of two GST genes through *trans* action.

### Lipid metabolism

Low temperatures alter the permeability of biofilms and stabilize weak chemical bonds, forcing the cell membrane to convert from a liquid phase to a thin-gel phase and thereby reducing membrane fluidity and affecting the functional properties of membrane proteins and membrane binding proteins [55, 56]. In insects, changes in membrane fluidity caused by cold temperatures can disrupt ion-water balance, causing muscle dysfunction, cold coma and death [57]. At low temperatures, plants, microorganisms and animals all increase the proportion of unsaturated fatty acids in the phospholipids that constitute cell membranes, because the changes in physical membrane properties caused by the increased unsaturation compensate for the order of hydrocarbons present inside cell membranes at low temperatures [58]. After cold acclimation, the expression levels of genes related to synthesis of cholesterol, fatty acid, and unsaturated fatty acid were significantly increased. Ultra-long chain fatty acids and long-chain fatty acids are essential components of cell membranes. Elongation protein of very long chain fatty acids and fatty acid desaturase are key rate-limiting enzymes for fatty acid chain desaturation and elongation [59]. Cold acclimation can significantly increase the proportion of unsaturated fatty acids in cell membranes of *Drosophila suzukii* [60] and fatty acid desaturase can improve the low temperature tolerance and low temperature germination rate of rice [33]. Therefore, the adverse effects of low temperature on membrane fluidity were alleviated by increasing the genes of unsaturated fatty acid synthesis in *P. canaliculata*. Compared with Ca0, DEGs in Ca24 were significantly enriched in linoleic acid metabolism and retinol metabolism pathways. These pathways contain *cytochrome P450 3A24* and *cytochrome P450 3A5*, which were down-regulated by 8.11 and 12.07 times respectively in Ca24. This reduced expression of *cytochrome P450 3A24* and *cytochrome P450 3A5* genes may minimize linoleic acid decomposition. It has been shown that the linoleic acid content of *Drosophila* larvae increases after cold acclimation [61], leading to a proposal that certain proportions of linoleic acid can maintain the fluidity at different temperatures [62]. The two members of *cytochrome P450* gene family mentioned above were regulated in *cis* by *lncRNA 112,557,458* and *lncRNA112569753*, respectively. We speculated that the decreased expression of *cytochrome P450*, may be a strategy to reduce the degradation rate of unsaturated fatty acids such as linoleic acid, which can maintain the balance of cell membrane fluidity at lower temperatures.

## Conclusion

In this study, transcriptome sequencing technology was used to investigate changes in protein coding genes and lncRNA expression in *P. canaliculata* after cold acclimation and cold stress. Genes related to cold tolerance and regulation of gene expression in *P. canaliculata* were identified. GO enrichment analysis showed that many DEGs were enriched in various metabolic processes. KEGG enrichment analysis showed that DEGs and many target genes of DELs were significantly enriched in proteasome, linoleic acid metabolism and retinol metabolism KEGG metabolic pathway. Furthermore, the lncRNA of *P. canaliculata* could participate in cold acclimation by regulating the expression of *E3 ubiquitin protein ligase*, *26S proteasome non-ATPase dependent regulation subunit*, *glutathione S-transferase, sodium/glucose cotransporter* and *cytochrome P450*.

## Methods

### Ethics approval and consent to participate

Ethical approval for this study was obtained from the Animal Research Ethics Committee of Nanjing Normal University. All the experimental procedures were approved by the Animal Research Ethics Committee of Nanjing Normal University.

### Sampling and treatment conditions

*P. canaliculata* were collected in June, 2020 from Lishui City, Zhejiang province, China. To eliminate confounding effects due to previous environmental differences, snails were reared in an artificial climate box for two weeks in the controlled climate chamber (photoperiod:16 L:8 D, illumination intensity: 2000 lx, relative humidity: 80%, temperature 25 °C). Lettuce and a few grains of carp food were provided daily as basic food. Aerated water was changed daily to remove the excrement and uneaten food debris.

Snails used in this study were then divided into two groups: the cold acclimation group and the control group (25 °C). There were three replicates with sample size of 30 snails with shell height of 28.84 ± 3.33 mm for each group, wrapped in a moist towel and confined in plastic containers (18 × 12 × 6 cm). Snails in the cold acclimation group were acclimated to cold starting at 25 °C, decreased by 5 °C every 5 d (at a rate of 1 °C a day) from 25 °C to 10 °C and continuously kept at 10 °C for four weeks [13]. Snails in the control group were not cold acclimated and treated at 25 °C during this period. After the cold acclimation, the cold acclimation group and the control group were exposed to cold treatment at 0 °C for 24 h in the same artificial climate box. No feeding was performed during the experiment. Hepatopancreas samples of three individuals were collected from the cold acclimation group and the control group after the cold acclimation ended and after 24 h cold stress at 0 °C, respectively. A total of 12 snails were sampled at two time points from the cold acclimation group (Ca0, Ca24) and the control group (Con0, Con24). Tissues were frozen immediately with liquid nitrogen and stored at − 80 °C for RNA extraction.

### RNA extraction, library construction, and RNA sequencing

Total RNA was extracted using an RNA kit (Novogene, Beijing, China) and qualified using a 1% agarose gel electrophoresis to assess possible contamination and degradation. RNA purity and concentration were examined using a NanoPhotometer® spectrophotometer. RNA integrity and quantity were measured using an RNA Nano 6000 Assay Kit with the Bioanalyzer 2100 system. RNA libraries for lncRNA-seq were prepared in a stranded manner with rRNA depletion. Briefly, the rRNA Removal Kit (RS-122–2402, Illumina, USA) was used to deplete the ribosomal RNA from total RNA following manufacturer’s instruction. Subsequently, the libraries were sequenced using the Illumina HiSeq 4000 by Novogene Co. Ltd (Beijing, China) and required reads (150 bp paired-end) were generated.

### Transcript assembly and identification of lncRNAs

All raw data were first filtered by Fastp [63] to eliminate the low-quality data reads and adaptor sequences. Q30 and GC content of clean data were calculated at this stem. All downstream analyses were based on clean data of high quality. Clean reads for each sample were mapped to the reference genome [38] using the software HISAT2 [64]. Reads alignment results were transferred to the program StringTie [65] for transcript assembly. All transcripts were merged using Cuffmerge software [66]. LncRNAs were then identified from the assembled transcripts according to the following four steps [67, 68]: (1) removal of low expression transcripts with fragments per kilobase per million fragments mapped (FPKM) < 0.5 [69]; (2) removal of short transcripts less than 200 bp and 2 exons; (3) removal of transcripts with protein-coding capability according to Coding Potential Calculator 2 (CPC2), Pfam and Coding Non-Coding Index (CNCI) databases [70–72]; (4) removal of transcripts mapped within 1 kb of an annotated gene as determined by Cuffcompare.

### Analysis of differentially expressed genes and lncRNAs

Quantifications of transcripts and genes were performed using StringTie software to obtain transcripts per million reads (TPM) values. EdgeR was used for differential expression analysis, and the resulting *P*-values were adjusted using the Benjamini and Hochberg’s approach [73] for controlling the false discovery rate. Genes with |log2 (fold change) |> 1 and *padj* < 0.05 were classified as differentially expressed.

### qRT-PCR analysis of gene expression

The qRT-PCR assays were performed to validate the consistency of RNA-Seq analysis. qRT-PCR was performed on 13 DEGs selected from the RNA sequencing data according to their potential functional importance, and DELs from Primer-BLAST (https://www.ncbi.nlm.nih.gov/tools/primer-blast/) was utilized in primer design, and primer pair specificity was determined through PCR product sequencing (Table S2). Total RNA was extracted from hepatopancreas of 12 snails (the same individuals used for transcriptome sequencing, three biological replicates were set for each treatment at 0 h and 24 h, respectively) according to the instructions of the RNA kit (Tiangen, Beijing, China). First strand cDNA was synthesized using a HiScript III 1st Strand cDNA Synthesis Kit (Vazyme, Nanjing, China). RT-qPCR was then performed on each sample in triplicate with SYBR qPCR Master Mix (High ROX Premixed, Vazyme, Nanjing, China) using the 7500 Fast Real-Time PCR System (Applied Biosystems™, Foster City, CA, USA) in a 20 μL reaction volume. The *β-actin* gene was used as an internal reference gene to normalize the gene expression levels [74]. The cycling parameters for the PCR amplification were as follows: 95 °C for 30 s, 40 cycles of 95 °C for 10 s, 60 °C for 30 s, followed by 1 cycle of 95 °C for 15 s, and 60 °C for 60 s. Amplification specificity was validated by melting curve analysis. The relative expression levels of target genes were calculated by the comparative cycle threshold (Ct) method (2 ^−ΔΔCt^) and subjected to statistical analysis with SPSS (versions 22.0).

### LncRNA target gene prediction

Target gene prediction for lncRNAs was carried out in two ways: the *cis*-acting target gene prediction (co-location analysis), and *trans*-acting target gene prediction (co-expression analysis). Based on the working model of *cis*-acting regulatory element activity, the protein coding genes located within 100 kb from the lncRNA of interest were selected as potential *cis*-acting targets. For *trans*-acting target prediction, the Pearson’ correlation coefficients (|*r*|> 0.95 and *p*-value < 0.05) between coding genes and lncRNAs were calculated and analyzed to identify *trans*-acting regulatory elements.

### GO and KEGG enrichment analyses

Analyses of GO and KEGG [75–77] enrichment for DEGs or DELs target genes were performed using the clusterProfiler [78] in R package, with corrections made for gene length bias. Enrichment was considered to be significant when the corrected q-values was less than 0.05.

### Availability of supporting data

Supplementary files Table S1 to S5 and Fig. S1 to S2 are available in the additional files.

## Supplementary Information

**Additional file 1: Supplementary Fig. S1.** Verification of the selected DEGs and DELs by qRT-PCR as compared with RNA-seq data. a: Ca0 vs Con0; b: Con24 vs Con0; c: Ca24 vs Ca0; d: Ca24 vs Con24. The gene marked red represents differentially expressed gene. 

**Additional file 2: Supplementary Fig. S2.** KEGG enrichment analysis of co-location target genes of DELs in cold acclimation group after cold stress (Ca24 vs Ca0). “Rich factor” means that the ratio of the number of the genes in the specific subcluster and the number of genes annotated in this pathway.

**Additional file 3: Supplementary Table S1.** Quality control (QC) information for mRNA sequences in Pomacea canaliculata. **Table S2**. The list of candidate genes forqRT-PCR validation in *Pomacea canaliculata*. **Table S3**. The 2^-^^△△^^Ct^ values of candidategenes for qRT-PCR validation in *Pomacea canaliculata*.

**Additional file 4: Table S4.** KEGG pathways with differentially expressed genes and target genes from differentially expressed lncRNAs in different groups

**Additional file 5: Table S5.** Significant GO classifications associated with target genes from differentially expressed lncRNAs and mRNAs in different groups

## Data Availability

Raw reads for the transcriptomic analysis have been uploaded on the SRA database from NCBI under the BioProject PRJNA788380 and will be available after publication.
