# Supplementary figures and images for: Transcriptome sequencing reveals the differentially expressed lncRNAs and mRNAs in response to cold acclimation and cold stress in Pomacea canaliculata
Source: BMC Genomics. 2022 May 19;23:382. doi: 10.1186/s12864-022-08622-5 (PMC9121591; doi:10.1186/s12864-022-08622-5)

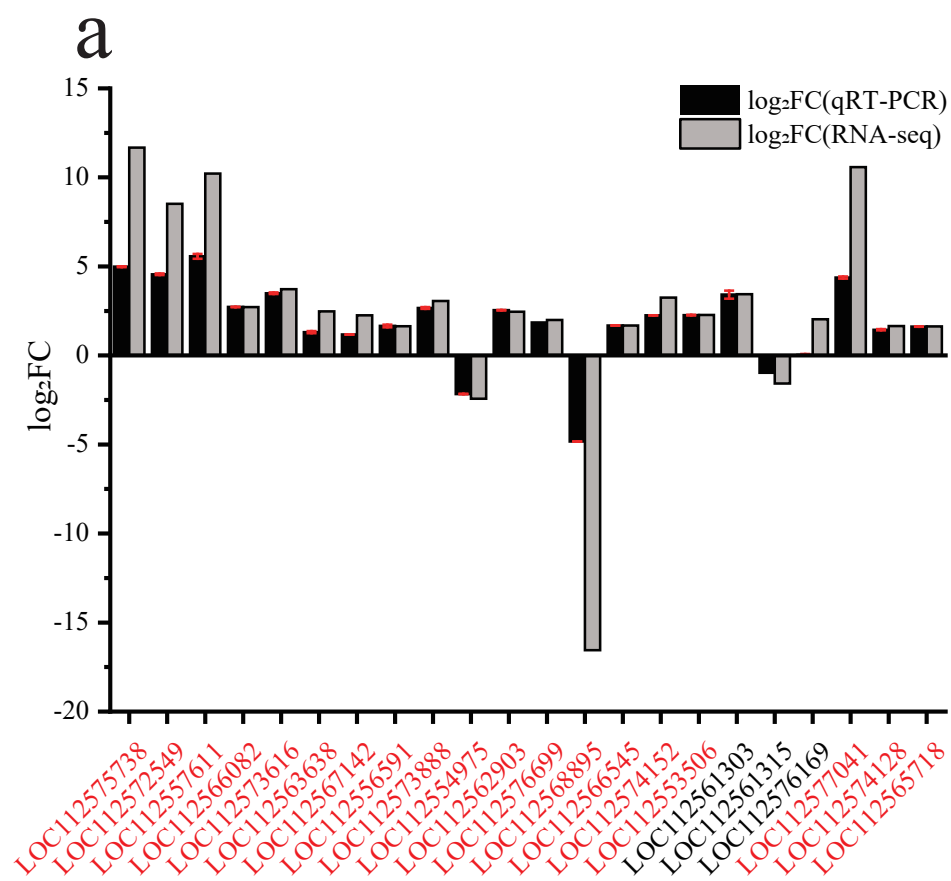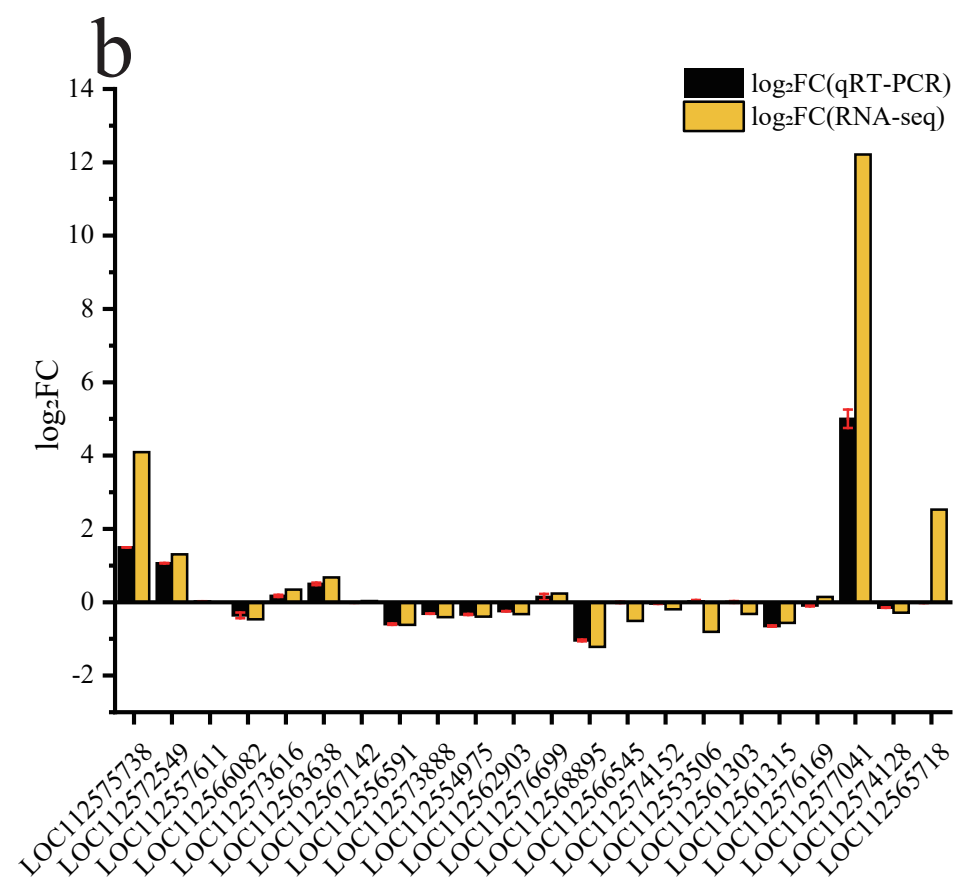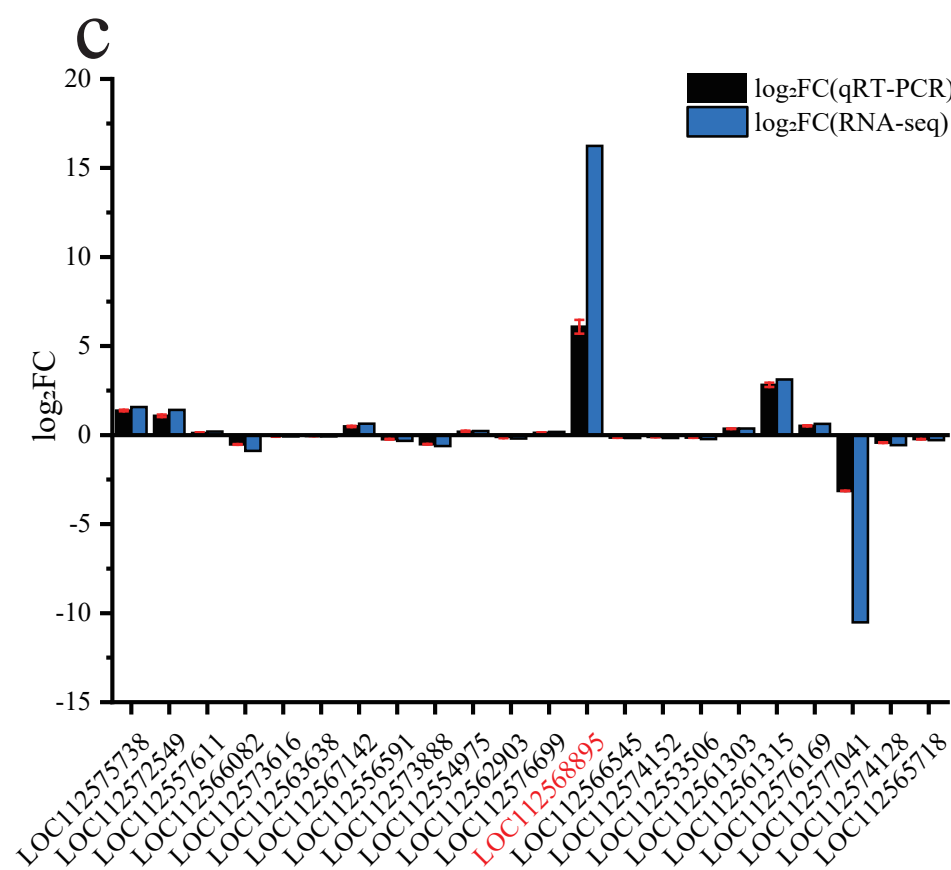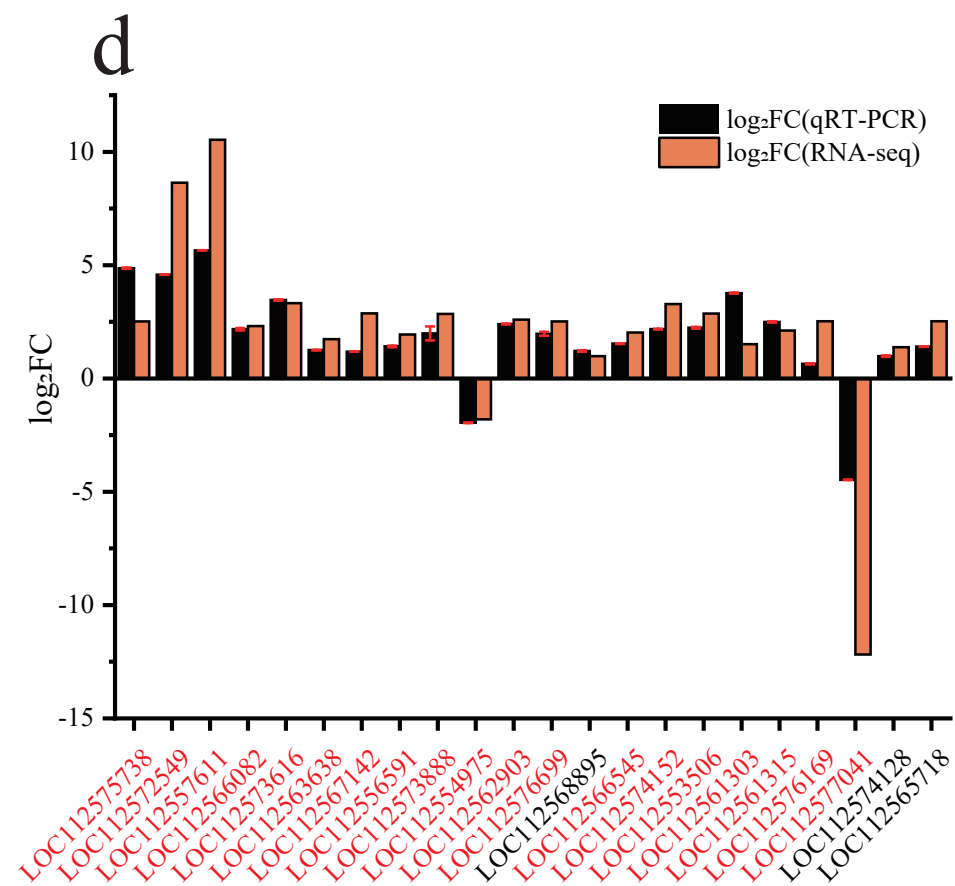

Supplement: Supplementary file 1 — Additional file 1: Supplementary Fig. S1. Verification of the selected DEGs and DELs by qRT-PCR as compared with RNA-seq data. a: Ca0 vs Con0; b: Con24 vs Con0; c: Ca24 vs Ca0; d: Ca24 vs Con24. The gene marked red represents differentially expressed gene. [file 12864_2022_8622_MOESM1_ESM.pdf]

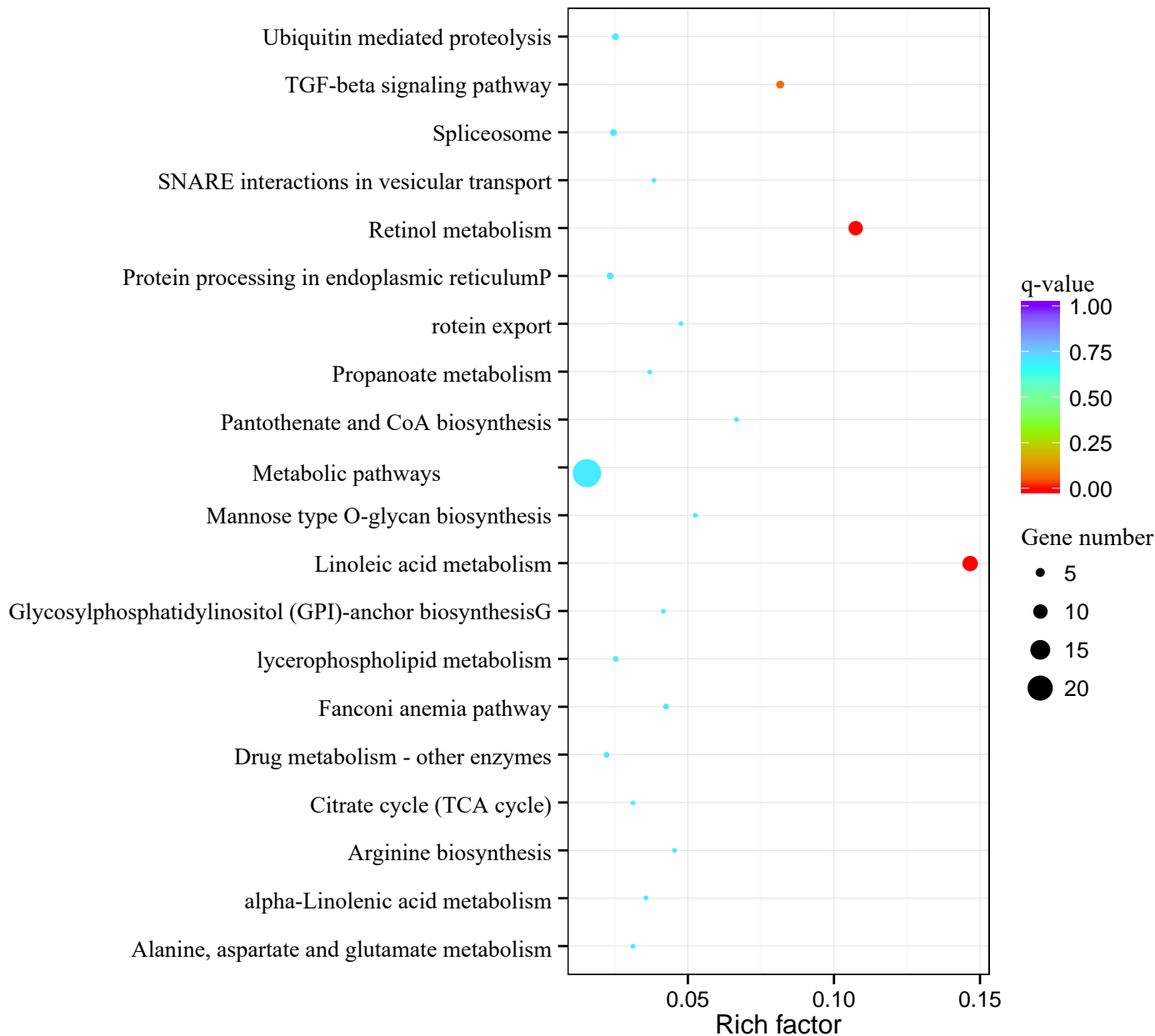

Supplement: Supplementary file 2 — Additional file 2: Supplementary Fig. S2. KEGG enrichment analysis of co-location target genes of DELs in cold acclimation group after cold stress (Ca24 vs Ca0). “Rich factor” means that the ratio of the number of the genes in the specific subcluster and the number of genes annotated in this pathway. [file 12864_2022_8622_MOESM2_ESM.pdf]
