# Supplementary material for: Transcriptome sequencing reveals the differentially expressed lncRNAs and mRNAs in response to cold acclimation and cold stress in Pomacea canaliculata
Source: BMC Genomics. 2022 May 19;23:382. doi: 10.1186/s12864-022-08622-5 (PMC9121591; doi:10.1186/s12864-022-08622-5)
Supplement: Supplementary file 3 — Additional file 3: Supplementary Table S1. Quality control (QC) information for mRNA sequences in Pomacea canaliculata. Table S2. The list of candidate genes forqRT-PCR validation in Pomacea canaliculata. Table S3. The 2-△△Ct values of candidategenes for qRT-PCR validation in Pomacea canaliculata. [file 12864_2022_8622_MOESM3_ESM.docx]

**Supplementary Table S1.** Quality control (QC) information for mRNA sequences in *Pomacea canaliculata*.

| **Sample group** | **Sample_name** | **Raw_bases (G)** | **Clean_bases (G)** | **Q30 value (%)** | **GC_content (%)** | **Total mapped (%)** |
| --- | --- | --- | --- | --- | --- | --- |
| Ca0 | Ca0-1 | 12.35 | 12.17 | 93.93 | 47.32 | 86.33% |
|  | Ca0-2 | 12.52 | 12.31 | 92.11 | 46.30 | 84.20% |
|  | Ca0-3 | 12.42 | 12.21 | 91.91 | 46.51 | 83.92% |
| Ca24 | Ca24-1 | 13.09 | 12.81 | 92.25 | 44.98 | 84.36% |
|  | Ca24-2 | 12.72 | 12.45 | 91.12 | 45.64 | 81.33% |
|  | Ca24-3 | 12.39 | 11.9 | 91.55 | 45.69 | 83.78% |
| Con0 | Con0-1 | 12.59 | 12.28 | 92.14 | 45.84 | 85.72% |
|  | Con0-2 | 12.57 | 12.34 | 92.35 | 47.68 | 87.00% |
|  | Con0-3 | 12.95 | 12.72 | 91.11 | 46.23 | 86.05% |
| Con24 | Con24-1 | 13.31 | 13.06 | 91.74 | 45.46 | 85.95% |
|  | Con24-2 | 12.44 | 12.28 | 91.85 | 45.95 | 85.57% |
|  | Con24-3 | 12.24 | 12.03 | 91.09 | 46.05 | 85.23% |

**Supplementary Table S2**. The list of candidate genes for qRT-PCR validation in *Pomacea canaliculata*.

| **Gene ID** | **Forward primer** | **Reverse primer** | **Annotations** |
| --- | --- | --- | --- |
| LOC112575738 | ACGATGAAGACGAATACCTGTGT | AACATCAGCAGTCCCTCTCG | *G-protein coupled receptor GRL101* |
| LOC112572549 | TTGTCAGTTTGAAGACGCACG | GGAAGCTGTCCGAAAGGAGT | *Glutathione S-transferase* |
| LOC112557611 | CATAAGGCTGTGCTCCGTGAC | TTCACAGCCTCATACTCCTCCTT | *GTPase IMAP family member 8* |
| LOC112566082 | ATATGCCGAGAGACGCTGC | ACGGACCTTGGTGTTTAGGG | *Sodium/potassium-transporting ATPase subunit beta-1* |
| LOC112573616 | GAACCGACTTGCTCGAAACC | CAGTTCAGCCTTACCAGCAGA | *F-box only protein 4* |
| LOC112563638 | CTGGATGTCTCGCTGCTGAAT | TCTAAAACCTGACCAAAACGCTC | *Heat shock 70 kDa protein 13* |
| LOC112567142 | GGACATCAGCAAGAACGCAC | GGGTCTTACGGAACAGGTCG | *Heat shock protein 70 B2* |
| LOC112556591 | AGTTGGTTGAGCAGCGAATG | CTGTGAGGCGTGGAAAGACC | *Myeloid differentiation primary response protein MyD88* |
| LOC112573888 | CAAGAACAGCTGGACCAACCC | CCAAACCAAGACATTCCGCC | *Elongation of very long chain Fatty acids protein 2* |
| LOC112554975 | CGACGGGTAAAAACAAGCCG | GGCGACCCAACAAACTCAAG | *Glycogenin-1* |
| LOC112562903 | CGTAGAAGCAGTGGACCCAG | ACCACCTTTCCTTTGACGCT | *26S proteasome non-ATPase regulatory subunit 14* |
| LOC112576699 | TGGAGCCAACTGTGGTAGGA | AAAGGTGCTCGTCAACCTCA | *E3 ubiquitin protein ligase TRAF7* |
| LOC112568895 | TATATTGCTGCCTTGGGAGGG | CTCCACTGTCCTCACATCTCC | *Intersectin-1* |
| LOC112566545 | CAAAAGAGAGGCGAAGGAGC | GGTCAGATGTACGGAGGGTG | LncRNA |
| LOC112574152 | AAAAGTGCCCTGGGACTGAAT | TTGAACCTTGGCACTTGGCT | LncRNA |
| LOC112553506 | AGTGTAGGAATGACTGGGGCA | AGCCTCCGAAGTTAAAGCGA | LncRNA |
| LOC112561303 | TGCCAGCAAGCTTCAGTAACC | CGGACCTTATTCACCAGCGA | LncRNA |
| LOC112561315 | TTATGATTTCGTTTGAGGGGAGGA | CCCTCTCTGTACCTAAACCCA | LncRNA |
| LOC112576169 | CCTGGGATAAGCCAAATGCC | AAGGGCCCATGTGTCACTAC | LncRNA |
| LOC112577041 | AGAGGTTGAACGGCACACAC | GGCGAGAGCGATAAGTAGCA | LncRNA |
| LOC112574128 | ATACGTGTCTGTCCCGCTTTG | GTGGTCGGGGCAAGGAGTTA | LncRNA |
| LOC112565718 | GCTGACGTCACACCGAAACA | GTTCTTCGTTTGCACGCTTG | LncRNA |
| β-actin | TCACCATTGGCAACGAGCGAT | TCTCGTGAATACCAGCCGACT | β-actin |

**Supplementary Table S3**. The 2^-△△Ct^ values of candidate genes for qRT-PCR validation in *Pomacea canaliculata*.

| Gene ID | 2^-△△Ct^ | | | |
| --- | --- | --- | --- | --- |
|  | Ca0vsCon0 | Ca24vsCon24 | Con24vsCon0 | Ca24vsCa0 |
| LOC112575738 | 31.64±0.073 | 29.15±0.075 | 2.82±0.001 | 2.60±0.002 |
| LOC112572549 | 23.58±0.054 | 23.93±0.114 | 2.09±0.008 | 2.12±0.005 |
| LOC112557611 | 47.39±0.167 | 50.41±0.165 | 1.02±0.005 | 1.08±0.003 |
| LOC112566082 | 6.60±0.015 | 4.52±0.046 | 0.78±0.078 | 0.69±0.693 |
| LOC112573616 | 11.27±0.054 | 11.03±0.054 | 1.13±0.029 | 0.98±0.005 |
| LOC112563638 | 2.47±0.032 | 2.38±0.059 | 1.41±0.034 | 0.97±0.024 |
| LOC112567142 | 2.26±0.008 | 2.27±0.064 | 0.99±0.006 | 1.40±0.028 |
| LOC112556591 | 3.15±0.096 | 2.67±0.053 | 0.66±0.020 | 0.85±0.017 |
| LOC112573888 | 6.35±0.066 | 3.97±0.042 | 0.80±0.004 | 0.70±0.007 |
| LOC112554975 | 0.18±0.006 | 0.26±0.005 | 0.79±0.013 | 1.14±0.011 |
| LOC112562903 | 5.82±0.020 | 5.28±0.026 | 0.85±0.002 | 0.93±0.004 |
| LOC112576699 | 3.61±0.131 | 3.93±0.073 | 1.10±0.087 | 1.09±0.020 |
| LOC112568895 | 67.84±0.001 | 2.32±0.035 | 0.49±0.023 | 34.46±0.07 |
| LOC112566545 | 3.21±0.007 | 2.91±0.010 | 1.00±0.017 | 0.90±0.003 |
| LOC112574152 | 4.76±0.005 | 4.52±0.010 | 0.98±0.012 | 0.93±0.002 |
| LOC112553506 | 4.80±0.018 | 4.72±0.055 | 1.02±0.038 | 0.91±0.012 |
| LOC112561303 | 11.35±0.225 | 13.60±0.032 | 1.00±0.025 | 1.20±0.003 |
| LOC112561315 | 0.51±0.018 | 5.64±0.009 | 0.64±0.016 | 7.06±0.011 |
| LOC112576169 | 1.03±0.044 | 1.56±0.038 | 0.94±0.025 | 1.43±0.035 |
| LOC112577041 | 10.38±0.060 | 0.07±0.042 | 32.07±0.251 | 0.23±0.004 |
| LOC112574128 | 2.72±0.036 | 1.98±0.019 | 0.90±0.007 | 0.74±0.007 |
| LOC112565718 | 3.07±1.619 | 2.64±0.023 | 0.99±0.009 | 0.85±0.007 |
